# Impact of Sex Hormones on Glioblastoma: Sex-Related Differences and Neuroradiological Insights

**DOI:** 10.3390/life14121523

**Published:** 2024-11-21

**Authors:** Jessica Rossi, Marialuisa Zedde, Manuela Napoli, Rosario Pascarella, Anna Pisanello, Giuseppe Biagini, Franco Valzania

**Affiliations:** 1 Clinical and Experimental Medicine PhD Program, University of Modena and Reggio Emilia, 41121 Modena, Italy; jessica.rossi@ausl.re.it; 2 Neurology Unit, Stroke Unit, Azienda Unità Sanitaria Locale-IRCCS di Reggio Emilia, Viale Risorgimento 80, 42123 Reggio Emilia, Italyfranco.valzania@ausl.re.it (F.V.); 3 Neuroradiology Unit, Azienda Unità Sanitaria Locale-IRCCS di Reggio Emilia, Viale Risorgimento 80, 42123 Reggio Emilia, Italy; manuela.napoli@ausl.re.it (M.N.); rosario.pascarella@ausl.re.it (R.P.); 4 Department of Biomedical, Metabolic and Neural Sciences, University of Modena and Reggio Emilia, 41121 Modena, Italy; gbiagini@unimore.it

**Keywords:** glioblastoma, neurosteroids, androgen receptor, targeted therapy, MRI, DCE, PWI, MRS

## Abstract

Glioblastoma (GBM) displays significant gender disparities, being 1.6 times more prevalent in men, with a median survival time of 15.0 months for males compared to 25.5 months for females. These differences may be linked to gonadal steroid hormones, particularly testosterone, which interacts with the androgen receptor (AR) to promote tumor proliferation. Conversely, estrogen (E2), progesterone (P4), and P4 metabolites exert more complex effects on GBM. Despite these insights, the identification of reliable hormonal tumor markers remains challenging, and studies investigating hormone therapies yield inconclusive results due to small sample sizes and heterogeneous tumor histology. Additionally, genetic, epigenetic, and immunological factors play critical roles in sex disparities, with female patients demonstrating increased O6-Methylguanine-DNA methyltransferase promoter methylation and greater genomic instability. These complexities highlight the need for personalized therapeutic strategies that integrate hormonal influences alongside other sex-specific biological characteristics in the management of GBM. In this review, we present the current understanding of the potential role of sex hormones in the natural history of GBM.

## 1. Introduction

Glioblastoma (GBM) is the most aggressive and malignant primary brain tumor, classified as Grade 4 according to the World Health Organization (WHO) [1]. The standard treatment for GBM involves surgical resection followed by adjuvant radio-chemotherapy, commonly known as the Stupp protocol [2]. Despite this approach, survival for GBM patients remains poor. Therefore, identifying new therapeutic targets is crucial. Over the past decade, the discovery of novel prognostic and therapeutic biomarkers has deepened the understanding of GBM and highlighted its inherent heterogeneity. Molecular biomarkers, including MGMT methylation, IDH1 and IDH2 mutations, and 1p/19q codeletion, are routinely used for tumor classification in clinical settings.

Recent evidence highlights that the central nervous system (CNS) is capable of synthesizing gonadal steroid hormones from cholesterol, with those produced within the CNS known as neurosteroids. Neurosteroids are able to influence neuronal and glial cells, and both types of cells can synthetize them in the CNS and in the peripheral nervous system [3]. On the other hand, steroid hormones may contribute to the development of brain tumors, as their receptors belong to a superfamily of ligand-activated transcription factors with oncogenic potential [4]. Despite evidence suggesting their involvement in GBM pathogenesis, biomarkers related to steroid receptors are not yet part of standard clinical practice. 

In this review, we present the current understanding of the potential role of sex hormones in the development of GBM.

## 2. Biology and Epidemiology 

Glioblastoma is the most common malignant tumor of the CNS in adults, accounting for 82% of cases of malignant glioma and 45.6% of primary malignant brain tumors globally [5,6]. The incidence of GBM increases with age from 0.15 per 100,000 in children to the peak of 15.03 per 100,000 in patients aged 75–84 years [6,7]. Men are 1.6 folds more likely to be diagnosed with gliomas than women [8].

The majority of GBM cases are sporadic. However, some familial tumor syndromes could be associated with GBM, including tuberous sclerosis, neurofibromatosis type I, Li–Fraumeni syndrome, ataxia-telangiectasia, Lynch syndrome, and Turcot syndrome [8,9]. In sporadic cases, no distinct carcinogenic causes have been identified to date. The only well-established risk factor is exposure to high-dose ionizing radiation, with an estimated 2.5% overall risk of developing GBM following radiotherapy [10,11,12]. No definitive link has been demonstrated between GBM and environmental factors such as smoking, dietary risks, cell phone use, electromagnetic fields, severe head trauma, occupational exposures, or pesticide contact [11,12]. Protective factors include atopic conditions (e.g., asthma, eczema, hay fever, and allergies), which reduce the risk of gliomas by approximately 30%, as well as the long-term use of low-dose aspirin [13,14,15].

It is hypothesized that adult GBM may arise from a restricted pool of adult neural stem cells and progenitor cells located in specific brain regions, including the subventricular zone (SVZ), subcortical white matter, and the dentate gyrus of the hippocampus [6]. Especially the SVZ, situated along the lateral wall of the lateral ventricle, is a key site in the adult brain where neural stem cells and astrocyte precursors reside [16]. During neural development, these stem cells migrate from the SVZ and differentiate into various progenitor cells, positioning themselves at different distances from the SVZ [16]. 

Glioblastoma development involves a multistep process characterized by sequential and cumulative genetic alterations, as well as dysregulated growth factor signaling pathways influenced by both intrinsic and environmental factors, leading to malignant transformation [5,17]. This is orchestrated by various molecular factors, including the epidermal growth factor receptor (EGFR), vascular endothelial growth factor (VEGF), platelet-derived growth factor (PDGF), hepatocyte growth factor (HGF), and loss of phosphatase and tensin homolog (PTEN) [17,18].

As of the 2021 WHO Classification of Central Nervous System Tumors (fifth edition), GBM now exclusively refers to isocitrate dehydrogenase (IDH) wildtype tumors. Isocitrate dehydrogenase (IDH) hotspot mutations typically affect genes encoding either cytoplasmic IDH1 (90%) or mitochondrial IDH2 (10%) enzymes [19]. These mutations alter the normal conversion of isocitrate to alpha-ketoglutarate (α-KG), instead leading to the accumulation of the “oncometabolite” D-2-hydroxyglutarate (D-2HG). Due to its structural similarity to α-KG, D-2HG acts as a competitive inhibitor of various histone and DNA demethylases [20]. This inhibition triggers epigenetic reprogramming, characterized by extensive DNA hypermethylation and an ensuing block in cell differentiation. This dedifferentiated state provides a permissive environment for further genetic abnormalities, ultimately promoting tumor development and progression [20]. Diffuse astrocytic tumors with IDH mutations are grouped into a single category, known as IDH-mutant astrocytoma, with WHO grades ranging from 2 to 4 [21]. This differentiation between IDH-wildtype and IDH-mutant tumors marks significant progress in GBM classification. However, the literature published before 2021 is based on the 2016 WHO classification (or earlier versions), where the term “GBM” includes not only IDH-wildtype GBM but also the newly classified Grade 4 IDH-mutant astrocytoma [22].

## 3. Neuroradiological Pattern 

### 3.1. Conventional MRI

Brain MRI with conventional imaging sequences is essential for diagnosing and monitoring GBM. The traditional sequences used in neuro-oncology include T1-weighted (T1W), T2-weighted (T2W) and T2-weighted fluid attenuation inversion recovery (FLAIR) and post-contrast T1-weighted (T1W + c) imaging [23]. These sequences provide detailed anatomical information about the brain and the tumor, allowing for the assessment of peritumoral edema and blood–brain barrier (BBB) disruptions. Glioblastomas typically display a heterogeneous appearance on T1W and T2W images due to necrosis, hemorrhage, soft-tissue mass, and tumor vasculature. The presence of an irregular enhancing lesion with infiltrating tumor areas and cortical expansion is strongly indicative of GBM. However, using conventional MRI sequences alone has its limitations. Distinguishing GBM from other intracerebral masses with cystic or necrotic components (such as other neuroglial tumors, brain metastases, or brain abscesses) can be challenging. Furthermore, conventional MRI has a limited ability to differentiate between high- and low-grade gliomas. In GBM, conventional MRI sequences can reveal key features that help predict molecular markers. For instance, the presence of small regions of enhancement, a larger non-enhancing tumor component, well-defined tumor margins, and T1-weighted hypointense areas with suppressed FLAIR signals within necrotic regions are indicative of an IDH1 mutation [23,24]. Additionally, a greater volume of T2-weighted abnormalities and a higher T2W-to-T1W + contrast ratio of tumor components have also been linked to IDH1 mutations [25]. Studies have frequently reported that GBMs with IDH1 mutations are most commonly located in the frontal lobe [26,27]. 

Apart from IDH, the O6-Methylguanine-DNA methyltransferase (MGMT) gene is another extensively studied marker in GBM. The methylation status of MGMT is a crucial biomarker, as high MGMT activity (i.e., unmethylated MGMT) is associated with a decreased effectiveness of alkylating chemotherapy agents like temozolomide. In high-grade gliomas such as GBM, MGMT methylation is less frequent compared to low-grade gliomas [28]. In conventional MRI, tumors with hypermethylated MGMT often exhibit mixed-nodular enhancement in lesions that are not located in the temporal region [29]. Conversely, gliomas with unmethylated MGMT typically display a ring-pattern enhancement [30].

In non-GBM tumors, two specific radiological signs in conventional MRI sequences have been identified that can provide insights into their mutational status. The first is the “T2-FLAIR mismatch sign”, characterized by areas of high signal intensity on T2-weighted images that appear to be relatively hypointense on T2-FLAIR images due to incomplete free water suppression. Additionally, a rim of hyperintensity can often be seen in FLAIR images. This radiogenomic signature is considered a strong indicator of diffuse astrocytoma (IDH-mutant, 1p/19q intact), with high positive predictive power [31]. The second radiogenomic signature concerns the delineation of the T2-weighted hyperintense signal from normal brain parenchyma. When the hyperintense area shows smooth borders and a homogeneous signal intensity, the tumor is more likely to be an astrocytoma without 1p/19q co-deletion [32].

After surgical intervention, MRI should ideally be performed within 2 days to evaluate the extent of resection, check for a residual tumor, and identify any post-surgical complications [33]. In this post-operative context, blood products in the resection cavity may appear similar to residual enhancing lesions due to their intrinsic T1-shortening effects. Therefore, pre- and post-contrast T1-weighted images must be carefully interpreted. Enhancing lesions with a nodular appearance typically indicate residual neoplasm, but this is not always definitive. Different treatments, including chemotherapy and radiation, can affect vascular permeability, potentially causing new enhancing lesions. If this contrast enhancement is due to treatment-induced vascular leakage, it is termed pseudoprogression (PsP). In contrast, enhancement indicating tumor recurrence is referred to as tumor progression (TP). 

Distinguishing between PsP and TP using conventional MRI alone is challenging. A 2011 study evaluated 11 signs visible in conventional MRI to differentiate TP from PsP [34]: new enhancement, marginal enhancement around the surgical cavity, nodular enhancement, callosal enhancement, subependymal enhancement, spreading the wavefront of enhancement, cystic or necrotic change, increased peritumoral T2 abnormality, diffusion restriction, decreasing enhancement intensity, and increasing cystic or necrotic change. Of these, only subependymal enhancement showed a limited predictive power, with a 38% sensitivity, a 93% specificity, and a 42% negative predictive value. The remaining 10 signs were found to have no predictive value in distinguishing between PsP and TP.

### 3.2. Advanced MRI

Diffusion-weighted imaging (DWI) is a technique based on the random Brownian motion of water molecules, with the magnitude of this motion estimated as the apparent diffusion coefficient (ADC; mm^2^/s). The ADC value is influenced by cellular density and the presence of macromolecules, organelles, or cell membranes within tissue compartments [35]. In gliomas, there is an inverse correlation between ADC values and tumor grade, with lower ADC values typically indicating higher-grade tumors [36]. DWI has several applications in glioma management. It can help detect early tumor recurrence in both enhancing and non-enhancing lesions, often indicated by reduced diffusion [37], and can also be used to predict overall survival and progression-free survival in patients with GBM. Additionally, DWI has been proposed as a method to identify MGMT methylation status, with studies suggesting that a median minimum ADC (ADC_min_) value of 800 × 10^−6^ mm^2^/s or higher is indicative of a methylated MGMT status [38,39]. However, DWI is most commonly employed to distinguish brain abscesses from gliomas and to identify ischemic areas in the post-operative phase.

The differentiation between PsP and tumor progression (TP) based on ADC values has been investigated extensively. In general, PsP lesions tend to exhibit a higher mean ADC value compared to TP lesions [37]. However, meta-analyses in this area are challenging due to the variation in reported ADC metrics across studies (e.g., mean, median, maximum, and minimum). For accurate quantitative assessments, it is recommended to use the mean ADC value of a region of interest (ROI), carefully excluding necrotic areas from the measurements. Reported cut-off values for the mean ADC to distinguish TP from PsP range between 1000 × 10^−6^ and 1412 × 10^−6^ mm^2^/s, with a corresponding sensitivity and specificity ranging from 78% to 98.3% and 63.6% to 100%, respectively [40,41]. The highest accuracy (a sensitivity of 98.3% and specificity of 100%) was reported using a cut-off mean ADC value of 1313 × 10^−6^ mm^2^/s, where higher values were indicative of TP. It is important to note that ADC values in post-treatment gliomas can be affected by several factors, including post-operative artifacts (e.g., pneumocranium) and MRI system-related specifications such as magnetic field strength and b-values. To account for these variables, it is recommended to perform ROI analysis at fixed locations over different time points to assess longitudinal changes in ADC values accurately.

Diffusion tensor imaging (DTI) models the complex diffusivity of water molecules within tissues, allowing for an evaluation of microstructural organization. In DTI, additional gradient pulses introduce a random phase shift for diffusing molecules while stationary molecules are canceled out [42]. Generally, water molecule diffusion in biological tissues is anisotropic, meaning that diffusion varies depending on direction. The diffusion tensor can be visualized as an ellipsoid, with its main axis oriented parallel to the principal diffusion direction within a voxel [42]. From the DTI model, various metrics can be derived, with the most used being mean diffusivity (MD) and fractional anisotropy (FA). MD is comparable to the apparent diffusion coefficient (ADC). FA serves as an index of diffusion anisotropy within the tissue: a value of 0 indicates isotropic diffusion (equal in all directions), while an FA value of 1 describes a maximally anisotropic voxel [43]. Some studies have shown that DTI metrics can assess the occult neoplastic invasion of white matter tracts and help predict the direction of tumor growth [44]. In clinical practice, DTI is primarily used for tractography to guide neurosurgical procedures.

In the context of differentiating PsP from tumor progression (TP), FA values have been found to be useful. Various studies, with a low or moderate risk of bias, have reported higher FA values in TP compared to PsP [45]. However, there are currently no prospective studies on this topic. Reported cut-off values for the mean FA to distinguish TP from PsP range between 0.13 and 0.18, with corresponding sensitivity and specificity values between 68% and 81% and 73% and 79%, respectively [46]. The highest sensitivity (81%) and specificity (79%) were reported using a cut-off FA value of 0.18, where lower FA values suggested TP [45]. Despite their utility, interpreting FA values faces limitations similar to those encountered with ADC-value interpretation. Factors such as post-operative artifacts, MRI system specifications, and the inherent variability in different regions of interest must be considered when analyzing DTI metrics.

Perfusion-weighted imaging (PWI) plays a significant role in GBM evaluation by assessing tumor neovascularization [47]. Neovascularization in tumors involves a complex network of poorly organized, leaky vessels with slow blood flow. In T1-weighted post-contrast images, these areas often show contrast enhancement, while the dynamic properties of the neo-angiogenic network can be further assessed using PWI. The most employed PWI techniques are dynamic susceptibility contrast (DSC) perfusion, dynamic contrast enhancement (DCE) perfusion, and arterial spin labeling (ASL).

DSC PWI is based on the signal loss induced in T2*-weighted sequences by a gadolinium-based contrast agent, bolus. The primary parameter in DSC is cerebral blood volume (CBV), which can be estimated and computed based on the negative enhancement integral. Other measurable parameters include cerebral blood flow (CBF), mean transit time (MTT), and time-to-peak (TTP). However, the area under the attenuation curve in DSC imaging only provides a proportional, not an absolute, CBV measurement. Therefore, CBV is expressed relative to a standard reference, usually the contralateral white matter, as the relative CBV ratio (rCBV ratio). The rCBV ratio serves as a robust indicator of hypervascular regions in GBM [48].

DCE PWI measures T1 shortening induced by a gadolinium-based contrast agent leaking from blood vessels into the surrounding tissue. Pharmacokinetic modeling derives multiple perfusion metrics, including the following: K_trans_, as representative of capillary permeability; V_e_, the fractional volume of the contrast agent in the extravascular–extracellular space; and V_p_, the fractional volume of the contrast agent in the plasma space. These parameters provide insight into the characteristics of the tumor microenvironment, especially in relation to its vascularity [49].

ASL is a contrast-free perfusion technique in which water molecules in blood vessels are magnetically tagged at the cervical level of the carotid artery before they enter a ROI, such as brain tissue. After a short interval (1.5–2.0 s), the labeled water molecules are imaged within the ROI, and CBF values are calculated from the signal differences between labeled and non-labeled images. The major advantage of ASL is that it is not affected by contrast leakage effects, making it a valuable tool in specific clinical scenarios [50].

PWI is also used to characterize glioma genotype, as genetic variations in glioma subtypes correlate with differences in tumor vasculature. For example, a recent review and meta-analysis reported that DSC-derived CBV values are fairly accurate in predicting IDH genotype, with an area under the receiver operator curve (AUROC) of 0.83 [51]. When reviewing DCE parameters, AUROCs of 0.81, 0.84, and 0.78 were reported for K_trans_, V_e_, and V_p_, respectively. Insufficient data exists regarding the non-invasive prediction of GBM genotype based solely on ASL perfusion metrics. However, some evidence suggests that ASL can differentiate between glioma grades (Grade 2, 3, 4) [52]. Additionally, studies have proposed that pre-treatment rCBV_max_ values can serve as a prognostic marker for overall survival or response to anti-angiogenic therapy in GBM [53].

PWI is most used in the post-therapeutic setting to differentiate between TP and pseudoprogression (PsP) in GBM patients. A meta-analysis examined the diagnostic accuracy of two DSC parameters: rCBV_mean_ and rCBV_max_ [54]. For rCBV_mean_, the pooled sensitivity and specificity for detecting TP were both 88% across a ratio threshold range of 0.9 to 2.15. For rCBVmax, the pooled sensitivity and specificity were 93% and 76%, respectively, with thresholds ranging from 1.5 to 3.1. 

DCE-PWI has been used to differentiate between TP and PsP in GBM patients. Recent meta-analyses report pooled sensitivity values for DCE-PWI ranging from 89% to 92%, with a specificity of 85% [54,55]. However, most studies included were not prospective, and the meta-analyses evaluated the overall diagnostic accuracy rather than specific parameters like K_trans_, V_e_, or V_p_. As a result, no threshold ranges are available for these DCE parameters.

ASL is suggested to be less accurate than other PWI techniques for differentiating PsP from TP. A recent meta-analysis found sensitivities between 52% and 79% and specificities between 64% and 82% for ASL [54]. Due to the limited number of studies, further research is needed. However, a recent study from our group indicates that ASL and DSC have similar diagnostic accuracies, suggesting that ASL could serve as an alternative to DSC-PWI. 

Magnetic resonance spectroscopy (MRS) is used to evaluate the chemical composition of tissue by detecting specific metabolites within defined regions or voxels. For GBM imaging, key metabolites include choline (Cho) and N-acetylaspartate (NAA). MRS can be performed using single-voxel techniques or multi-voxel approaches (chemical-shift imaging), but these methods may suffer from sampling errors and heterogeneous tumor content. Recent advancements such as 3D-echo planar spectroscopic imaging (3D EPSI) offer improved metabolic mapping with excellent spatial resolution and can be co-registered with anatomical images. MRS and 3D EPSI are valuable for assessing GBM metabolism and differentiating TP from PsP [56,57]. In GBM, elevated Cho levels are associated with an increased cell density and cell membrane content, while reduced NAA levels indicate decreased neuronal viability. Thus, an increased Cho/NAA ratio suggests TP, although Cho levels may also be elevated in patients undergoing immunotherapy. [58]

A meta-analysis of 55 studies found that MRS was superior to other MRI sequences (conventional, ADC, DSC PWI, and DCE PWI) for distinguishing PsP from TP, with a pooled sensitivity of 91% and specificity of 95% [55]. However, the meta-analysis included a mix of studies with single-voxel and multi-voxel MRS protocols and did not distinguish between the diagnostic capacities of different metabolite ratios. Reported cut-off values for metabolite ratios varied: Cho/Cre (1.07–2.50), Lac/Cho (1.05), and Cho/NAA (1.71). Further research is needed with standardized protocols and cut-off values.

**Figure 1:**
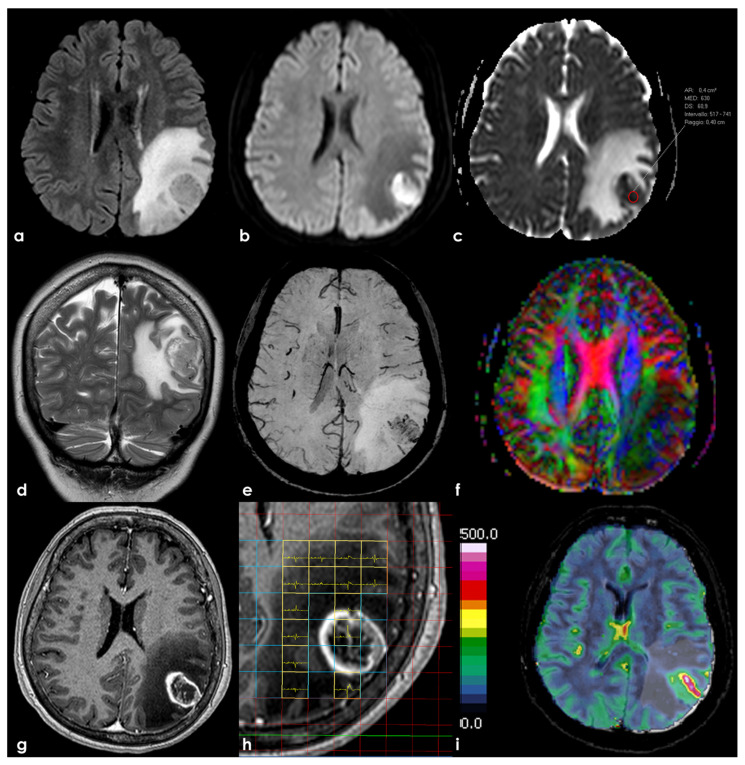
Brain MRI at the diagnosis of a female patient with GBM G4 WHO, MGMT methylated, IDH-wildtype, at the left temporal–parietal transition involving the angular gyrus. Panel a shows an axial FLAIR sequence with a mildly hypointense nodular lesion (with strongly hypointense linear structures within) surrounded by a wide area of hyperintense cytotoxic edema, expressed by a hypointense signal in the DWI (**b**) and hyperintensity in the ADC map (**c**). Conversely, the nodular neoplastic lesion is hyperintense in the DWI and hypointense in the ADC map. In (**d**), the coronal T2W sequence shows a similar finding as the FLAIR (**a**) with greater evidence of a dysomogenous signal intensity with hyperintensities within a hypointense signal in the nodular lesion. SWI (**e**) shows linear hypointensities within the nodule corresponding with vascular structures. The FA map (**f**) highlights the distortion and invasion of the pathways surrounding the left angular gyrus. The post-gadolinium T1W axial sequence (**g**) shows a peripheral ring-shaped contrast enhancement with mildly enhanced areas within the nodule. Multivoxel MRS (**h**) shows an increased Cho peak within the nodule and in the surrounding area of abnormal signal. The perfusional study (**i**) shows increased rCBV values in the lesional areas of contrast enhancement.

Recent studies have explored the prognostic value of MRS in GBM [59]. One study found that higher Cho/NAA ratios in the post-operative peritumoral edema zone are associated with early tumor recurrence and poorer prognosis, though this finding needs further validation. MRS can also help determine IDH mutation status by detecting elevated levels of 2-hydroxyglutarate, an oncometabolite associated with IDH-mutant gliomas [60]. A meta-analysis reported that MRS has a pooled sensitivity of 84% and a specificity of 97% for predicting IDH mutation status in GBM [61]. While promising, MRS is ideally performed at 3T or higher, requires expert interpretation or advanced software, and is less widely available compared to other MRI techniques. An example of an MRI study of a female patient with GBM is in Figure 1.

Sex differences in GBM have been observed across various aspects of diagnosis and progression, and neuroradiological features provide valuable insights into these disparities. Some studies indicate that men and women may exhibit sex-based differences in GBM imaging characteristics, particularly regarding the tumor location, volume, and patterns of growth. Men with GBM often present with larger tumor volumes, as well as more expansive regions of enhancement, necrotic cores, and peritumoral edemas compared to women. Additionally, GBM in males tends to frequently involve the temporal lobes, suggesting distinct spatial distribution patterns that could be influenced by underlying biological mechanisms associated with sex differences [62]. Moreover, GBM in men frequently involves both the right and left temporal lobes, suggesting a distinct spatial pattern of tumor localization and spread compared to that in women [62]. 

Sex-based differences in treatment response and radiological progression patterns are also being explored. Men often exhibit higher rates of early tumor recurrence visible on follow-up scans, which could correlate with lower overall survival rates compared to women [63]. These differences underscore the need for personalized imaging and treatment approaches in GBM, considering sex as a significant biological variable in neuroradiological assessment and therapeutic planning.

## 4. Sex Hormones: Mechanisms of Signaling and Their Role in Cancer Development

Sex hormones like estrogens, androgens, and progesterone operate primarily through genomic and non-genomic signaling mechanisms. After entering the cell, they bind to their specific receptors that, once activated, function as nuclear transcription factors. They translocate to the nucleus and bind to DNA, modulating the expression of various genes involved in cell proliferation, differentiation, and apoptosis [64]. Sex hormones also engage in rapid signaling processes by binding to membrane-associated receptors or by activating G protein-coupled receptors (GPCRs). This initiates secondary messenger cascades, such as the MAPK and PI3K/AKT pathways, that can impact cell behavior without directly involving gene transcription. For example, G protein-coupled estrogen receptors (GPERs) initiate rapid signaling responses that are crucial for cell migration and survival in several cell types, including cancer cells [65]. These non-genomic pathways add complexity to the regulatory network of sex hormones, influencing cellular responses in ways that complement genomic signaling. These processes often converge on the epidermal growth factor receptor (EGFR), amplifying its activity in various cellular contexts, including cancer. For instance, steroids can bind to GPERs, triggering intracellular signaling cascades that result in EGFR transactivation [66]. This is achieved through mechanisms like the release of ligands such as TGFα or the activation of kinases like Src, which phosphorylate the EGFR directly [67]. GPCRs, including GPERs, play a central role in mediating these effects by acting as upstream modulators. Through signaling intermediates like β-arrestins or direct interactions with adaptor proteins, GPCR activation leads to the engagement of downstream pathways such as the PI3K/AKT and MAPK/ERK pathways. These cascades promote critical processes like cell survival, migration, and proliferation, particularly in oncogenic settings. Furthermore, the interplay between GPERs and the EGFR exemplifies a functional crosstalk that enhances cellular responses, often creating positive feedback loops that sustain and amplify oncogenic signaling. This dynamic interaction adds complexity to the regulatory network of steroid hormones and underscores their potential role in tumor progression and therapy resistance [68].

Sex hormones significantly impact cancer development and progression in both men and women, particularly in hormone-sensitive cancers (including prostate, ovarian, and breast cancers). Furthermore, evidence points out that certain cancers traditionally considered non-hormone-sensitive, such as gastric, colorectal, and kidney cancers, exhibit sex-based differences, with a higher incidence observed in male than female patients. Estrogens play a role in maintaining epithelial barriers and have been shown to inhibit the growth of specific cancer cell types, including renal cell carcinoma [69]. On the other hand, androgens promote renal carcinoma growth by upregulating pro-survival factors and enhancing angiogenesis [70]. The protective effect of estrogens is partly due to their influence on cellular pathways that enhance barrier integrity and regulate cell proliferation. In gastric cancer, for example, estrogens can activate estrogen receptor pathways that may lead to reduced tumor growth through apoptotic mechanisms, modulating factors such as caspase activity and Bcl-2 expression levels [71]. These pathways collectively help in preserving tissue function and limiting cancer cell expansion in certain contexts. Thyroid cancer, particularly papillary and follicular types, is one of the few cancers that show a significantly higher prevalence in women compared to men, with women being approximately 2.9 times more likely to develop the disease. This gender disparity is thought to be influenced by the role of estrogen, which has been shown to preferentially promote the expression of the estrogen receptor alpha (ERα) over estrogen receptor beta (ERβ) in thyroid cancer cells [72]. This selective activation leads to enhanced cell proliferation and tumor growth. 

## 5. Sex-Related Differences and Hormonal Mechanisms in Glioblastoma

Glioblastoma is 1.6 times more common in men than in women, regardless of age, socioeconomic status, or geographic location. Moreover, male patients exhibit a survival disadvantage compared to female patients, with a median survival of 25.5 in females and 15.0 months in males in population-based datasets [73]. This suggests that gonadal steroid hormones, specifically testosterone, might have a role in tumor development through interaction with the androgen receptor (AR), promoting cell proliferation and tumor progression. 

The AR is a nuclear receptor primarily activated by testosterone and dihydrotestosterone. In its inactive state, the AR is predominantly found in the cytoplasm. Upon the binding of an androgen steroid, the activated AR translocates to the nucleus, dimerizes, and binds to specific hormone response elements, thereby regulating gene expression [74]. Higher AR expression has been demonstrated in GBM biopsies compared to normal brain tissue [75,76], and GBM patients with higher AR activity have demonstrated a worse prognosis [77]. In addition, AR expression levels appear to be associated with the histological grade of glial tumors, being elevated in GBM compared to Grade 2 and 3 astrocytomas [78]. Recent evidence points out that testosterone enhances tumor cell proliferation, migration, and invasion through its active metabolite dihydrotestosterone (DHT). This effect can be reversed by treatment with finasteride and dutasteride, both of which are 5α-reductase inhibitors [79]. Androgen receptor activation has also been shown to induce changes in the immune microenvironment, promoting the infiltration of immunosuppressive regulatory T-cells and contributing to GBM immune evasion [80]. Furthermore, a growing body of evidence shows that AR activation is strictly linked to EGFR signaling in GBM cells, as it can also be achieved by ligand-independent signaling through the EGFR [75]. The ErbB family of receptor tyrosine kinases, which includes EGFR, HER2, HER3, and HER4, plays a critical role in cell proliferation, survival, and differentiation. In GBM, the EGFR is frequently overexpressed or mutated, contributing to tumor aggressiveness and therapy resistance. By inhibiting these receptors, particularly the EGFR, compounds like afatinib disrupt key signaling pathways (e.g., the PI3K/AKT/mTOR pathway) that support GBM cell survival. Enzalutamide, an FDA-approved AR inhibitor for prostate cancer, has been shown to reduce cancer stem cell populations and improve survival by 50% in an orthotopic patient-derived xenograft (PDX) model of GBM [81]. Combining enzalutamide with an ErbB inhibitor, such as afatinib, may therefore enhance the antitumor effect in GBM cells by jointly targeting AR and ErbB signaling pathways, though more data are needed to confirm the therapeutic impact of this approach [82].

Another explanation for the higher incidence of GBM in men, as well as their worse prognosis, is the protective role of estradiol (E2) in glioma growth and progression. In vivo studies have demonstrated that estrogen-treated animals (both male and female) survived longer than ovariectomized, untreated female rats [83]. On the other hand, recent studies have evidenced that progesterone (P4) is capable of stimulating GBM stem cell growth, as well as the infiltration and migration of astrocyte [84,85]. In addition, it has been reported that GBM cells are capable of metabolizing P4 to 5α-dihydroprogesterone (5α-DHP) via 5α-reductase and further converting it to allopregnanolone (3α-THP) through 3α-hydroxysteroid dehydrogenase (3α-HSD), involving the AKR1C1-4 enzyme family. Allopregnanolone is synthesized in various brain regions and promotes cell proliferation, migration, and cytoprotection. In GBM cell lines, 3α-THP activates the proto-oncogene c-Src, a non-receptor tyrosine kinase that serves as a central protein in multiple cell signaling pathways. This activation supports processes such as inflammation, cell survival, proliferation, migration, invasion, and resistance to treatment, thereby promoting increased malignancy and tumor cell growth, likely through mechanisms that bypass the classical P4 receptor [86]. Some studies argue that in GBM cell lines allopregnanolone can enhance the temozolomide effect by decreasing DPYSL3/S100A11 expression and inducing DNA damage [87]. However, neurosteroids, including dehydroepiandrosterone and 17β-estradiol, are synthesized in TMZ-resistant GBM and maybe contribute to the development of drug resistance, as demonstrated in human GBM cell lines. Furthermore, 17βestradiol has attenuated TMZ-induced cell death and reduced reactive oxygen species production by mitochondria by increasing the expression of superoxide dismutase 1/2, catalase, and the nuclear factor erythroid 2-related factor (NRF) [88]. 

Recent evidence highlights that the effects of E2, P4, and P4 metabolites, such as 3α-THP, on tumorigenesis are concentration-dependent and influenced by the specific receptors predominantly expressed by tumor cells. This can explain why women can experience tumor progression (especially of astrocytomas) during pregnancy, which is characterized by elevated levels of progesterone and estradiol. In vitro and in vivo studies have demonstrated the dose-dependent role of progesterone in tumor cells, consisting of pro-tumorigenic effects at lower doses and anti-tumorigenic properties at very high doses [4]. The E2 protumorigenic effects are mediated through the activation of Erα, whereas the activation of Erβ favor a protective role. Therefore, a low intratumoral concentration of ERβ receptors appears to be associated with an increased risk of tumor growth and progression [89,90]. However, while Erα has been identified in approximately one-third of low-grade tumors, its expression may diminish or be lost during tumor progression [91]. Several studies have reported very low levels of estrogen and progestin receptors in human GBM, whereas glucocorticoid and/or androgen receptors have been found in a higher proportion of GBM [92,93]. However, not all studies have confirmed these findings [94,95]. Recent evidence suggests that differences in AR expression between male and female patients are not quantitative but rather regional [96]. AR expression is higher in the enhancing tumor periphery and peritumoral areas compared to the tumor core. In women, this elevated AR expression is particularly pronounced in the peritumoral region [96]. This interesting finding further confirms the important role of the peritumoral microenvironment in the genesis and progression of GBM.

Given the role of sex–steroid hormones in GBM growth and progression, several hormonal agonists and antagonists have also been examined for their effects on glioma cells. Treatment with micromolar concentrations of 2-methoxyestradiol (2-ME), a metabolite of estradiol, has been found to induce apoptosis in human and rat glioma cell lines [97,98]. Moreover, 2-ME may also inhibit the angiogenesis through a hypoxia-inducible factor 1α-dependent mechanism [99]. Despite recent findings showing that high doses of intraperitoneally administered 2-ME were effective in an orthotopic rat glioma model, clinical trials have demonstrated its limited oral bioavailability, highlighting a challenge for its therapeutic application in humans [92,100,101].

Several other estrogenic compounds have been investigated for their effects on glioma cells. Genistein, an isoflavone that preferentially binds to ERβ and inhibits protein tyrosine kinases and topoisomerase II, suppresses DNA synthesis in human glioma cells in a dose-dependent manner [102]. Tamoxifen is a selective estrogen receptor modulator (SERM) primarily used for breast cancer, with both estrogenic and antiestrogenic properties. It has been shown to reduce glioma cell proliferation and induce apoptosis, even in ER-negative glioma cells [103]. Tamoxifen’s effects appear to depend on the specific ER isoform, tumor stage, and dosage, acting as a pure ERβ antagonist at certain promoters but exhibiting agonist activity through non-ER mechanisms [104]. Notably, tamoxifen can interact with protein kinase C (PKC) and calmodulin independently of estrogen receptors. Through PKC inhibition, tamoxifen impacts cell signaling networks involved in proliferation and apoptosis. Additionally, tamoxifen modulates calcium signaling by inhibiting calmodulin, affecting various cellular processes without directly engaging ER pathways. These mechanisms underscore tamoxifen’s complex role in cancer therapy, extending beyond classic ER-mediated effects [104]. However, tamoxifen’s antitumor potential in glioma treatment appears to stem from its inhibition of protein kinase C (PKC), a crucial enzyme involved in tumor growth and angiogenesis. Unlike the lower dosage used for breast cancer (10–20 mg/day), significantly higher doses (160–240 mg/day) are required for gliomas, which can lead to side effects such as blood clots, an increased risk of uterine cancer, and weight gain [105]. The efficacy of Tamoxifen in clinical studies varies. In phase II trials, it resulted in tumor regression or stabilization in 45% of recurrent glioma patients, with longer survival seen in those with grade III tumors [105]. When combined with chemotherapy or radiation, outcomes are mixed, as some studies have reported improved survival rates while others have shown a minimal benefit [106,107,108,109]. However, these studies involved a small number of patients with heterogeneous clinical, histological, and molecular characteristics.

In addition to estrogenic agents, the progestin and glucocorticoid receptor antagonist RU486 (mifepristone) has been shown to block the ability of progesterone to stimulate the growth, migration, and invasion of human astrocytoma cell lines [110]. Mifepristone’s therapeutic potential extends to blocking GBM stem cell proliferation and migration. Notably, glucocorticoids like dexamethasone, commonly used for high-grade glioma symptoms, may exacerbate tumor growth and resistance to temozolomide by upregulating MGMT expression [111]. In contrast, mifepristone reduces MGMT protein expression, potentially enhancing temozolomide efficacy by increasing temozolomide-induced DNA damage, apoptosis, and tumor cell death [112]. Additionally, mifepristone inhibits VEGF and P-gp expression, both key factors in glioma chemoresistance, leading to increased temozolomide concentrations in the brain and reduced tumor growth [113,114]. Clinical studies show that mifepristone crosses the BBB, offering palliative effects in brain tumors with minimal side effects, making it a promising candidate for GBM treatment [114,115]. Further research is needed to elucidate its full potential in combination therapies. Table 1 summarizes the main known effects of sex hormones on the development and progression of GBM, as well as principal hormone antagonists studied for their potential antitumor activity.

## 6. Sex-Related Differences: Beyond Hormonal Mechanisms

Recent studies indicate that sex differences in GBM incidence and prognosis are not solely due to hormonal factors but also involve the contributions of sex chromosomes. The presence of two X chromosomes in females offers a layer of genetic complexity, as certain genes on the second X chromosome escape X-inactivation, potentially providing a protective effect against tumor development [116]. This partial inactivation allows females to express a subset of genes, such as *ATRX*, *DDX3X*, and *KDM6A*, that can influence immune response, DNA repair, and cellular growth, all of which are crucial in tumorigenesis and could underlie some of the sex-specific differences in GBM [117].

In males, the Y chromosome is also implicated in cancer biology, as it carries genes that play a role in cell cycle regulation and apoptosis. The loss of portions of the Y chromosome (or entire copies in some cells) has been associated with increased cancer susceptibility and aggressive tumor progression [118]. However, a recent analysis of the genomic and transcriptomic data of 13 cancer types (in 2375 patients) did not find a loss of the Y chromosome in glioblastoma or glioma patients [119].

Additionally, differences in chromosomal composition and sex hormones between men and women lead to variations in immune responses, with women generally having more vigorous immune anti-tumor responses than men. This can influence disease progression and outcomes in GBM [120]. Glioma-associated macrophages and microglia (GAMs) play a crucial role in GBM by regulating tumor growth, invasion, and survival. However, the behavior of microglia is modulated differently in males and females, with estrogen exerting a pro-inflammatory effect in female microglia (inducing a coordinated immune response against the tumor) and an anti-inflammatory effect in male microglia [121]. Astrocytes, which are also crucial to the brain’s immune response, exhibit sexual dimorphism in their cytokine production, potentially impacting glioma development. Specifically, male astrocytes tend to produce higher levels of IL-1β, IL-6, and tumor necrosis factor α pro-inflammatory cytokines than their female counterparts, which could influence the growth rates of GBM. Moreover, male patients display elevated myeloid-derived suppressor cells (MDSC) levels, a heterogeneous group of cells that inhibit immune responses and are linked to tumor progression and poor survival [122]. Targeted therapies show sex-specific effects in mice, with males benefiting from anti-proliferative agents targeting mMDSCs and females responding to IL-1B blockades with canakinumab [122]. Sex differences are also influenced by T cell behavior, with males showing greater CD8+ T cell exhaustion and higher progenitor-exhausted T cell frequencies, partially linked to the X chromosome escape gene *Kdm6a* [123]. These differences impact tumor progression and response to immunotherapy, such as anti-PD-1 treatment.

Furthermore, metabolic changes play a crucial role in tumor survival and progression and may contribute to observed sex differences in brain cancers, including GBM. Sex differences in glucose utilization are critical for cancer cell growth, and recent studies have indicated that high glycolytic gene expression in men correlates with poor survival, while women with a similar expression survive longer, suggesting that glycolytic metabolites may stratify survival by sex in GBM [124]. Cancer cells often rely on altered metabolic pathways, including aerobic glycolysis, mitochondrial function, and fatty acid oxidation, to survive in challenging environments. Mitochondria show sex differences in enzyme activity, with females displaying higher activity in enzymes like citrate synthase and succinate dehydrogenase but also lower levels of reactive oxygen species (ROS) and oxidative damage compared to males [125]. Moreover, a growing body of evidence indicates that male GBM exhibits a greater dependency on glutamine metabolism compared to its female counterpart, which may contribute to the observed sex-based differences in tumor behavior and prognosis [126]. In males, GBM cells often display an elevated activity of glutaminase (GLS), the enzyme that converts glutamine to glutamate, fueling the tricarboxylic acid (TCA) cycle and supporting ATP production, the biosynthesis of macromolecules, and redox balance. This metabolic preference provides a steady supply of precursors necessary for rapid tumor growth and survival in a nutrient-depleted microenvironment [126].

Sexual dimorphism significantly influences GBM biology and gene expression, contributing to its higher prevalence in men. Sex differences in molecular subtypes, gene expression, and tumor suppressors like p53 and RB1 affect tumor progression, with male GBM showing faster growth and distinct molecular characteristics compared to female GBM [127]. Differences in cyclin-dependent kinase inhibitors (p16, p21, p27) and the higher expression of tumor suppressors in females further contribute to sex-specific tumorigenesis [125,128]. 

Moreover, epigenetic modifications, such as the methylation status of the O6-methylguanine-DNA methyltransferase (MGMT) promoter, show sex-specific effects on GBM progression and therapy response, with a hypermethylated MGMT promoter status in about 80% of female patients vs. only 27% in men. MGMT promoter hypermethylation is linked to an improved chemotherapy response, which may account for the generally better chemotherapy and radiotherapy outcomes observed in women with GBM compared to men [129]. Moreover, female GBM exhibits a higher degree of genomic instability, including aneuploidy and increased tumor mutational burden. Integrative proteomic and phosphoproteomic analyses have revealed sex-specific differences in protein expression and phosphorylation activities. Notably, males show enhanced EGFR activation, while female patients display the hyperphosphorylation of SPP1 [130]. 

**Figure 2:**
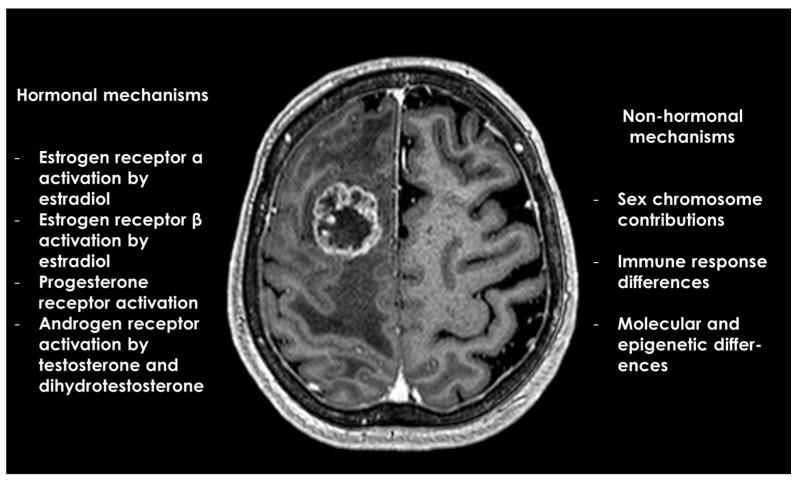
Summary of sex differences in glioblastoma: hormonal and non-hormonal mechanisms.

Table 2 and Figure 2 illustrate and summarize the main hormonal and non-hormonal mechanisms underlying sex differences in glioblastoma.

## 7. Discussion 

Glioblastoma is the most frequent malignant tumor of the CNS in the adult population and is characterized by an aggressive course and poor prognosis. Since the 2005 phase III trial by Stupp et al., which established the role of concurrent chemoradiation with temozolomide followed by adjuvant temozolomide for patients with newly diagnosed GBM, no other therapies tested in late-phase clinical trials have demonstrated significant improvement over this treatment approach [2]. 

A growing body of interest has focused on the therapeutic targeting of receptor tyrosine kinase (RTK) signaling pathways, as well as epigenetic modifications, metabolic pathways, and immune-targeted therapies [82]. These approaches aim to disrupt key mechanisms involved in tumor growth and survival, offering multiple strategies for potential therapeutic intervention. However, no therapies have significantly extended patient survival. Several factors contribute to treatment inefficacy, with the most important being notable intratumoral and intertumoral heterogeneity. Single-cell genetic analyses have shown that GBM cells can acquire new alterations over time, resulting in molecular profiles that differ from those identified during initial surgery [131]. Without re-surgery, therapies targeting the original molecular profile may become ineffective. Therapeutic pressure can also select subclones lacking the target molecule, further complicating treatment. It is becoming increasingly vital to adopt a multi-faceted approach to tumor targeting, addressing various molecular targets while considering the tumor’s specific characteristics and the unique profile of each patient.

Recent evidence suggests that regional and global differences in sex hormone concentrations between males and females with GBM may help explain the disparities in incidence and disease progression between sexes. These hormonal differences persist even during aging, as cerebral steroidogenesis continues into advanced age [132]. These variations in hormone levels can affect not only the risk of developing GBM but also how the disease manifests and progresses in male versus female patients. Nevertheless, specific hormonal tumor markers capable of predicting the behavior of GBM have yet to be identified. Additionally, studies investigating the role of hormone therapy in the treatment of GBM have not reached definitive conclusions. Many of these studies included a small number of patients or tumors with varying grades and histology, introducing significant biases. 

Furthermore, it has been observed that sex differences in the progression and incidence of GBM are not solely explained by hormonal mechanisms. Additional factors, such as genetic, epigenetic, and immunological differences between males and females, also play a role in driving these disparities. Recently, Jang et al. found that female GBM patients display increased MGMT promoter methylation [130], which is correlated with an increased overall survival and improved response to chemotherapy [2,133]. 

## 8. Conclusions 

These findings suggest distinct molecular mechanisms driving GBM in males and females, which may have important implications for the development of sex-specific therapeutic strategies. These complex factors influence treatment response and disease progression, highlighting the need for more personalized therapeutic strategies that consider not only hormonal influences but also other sex-specific biological characteristics. 

## Figures and Tables

**Table 1:** Summary of the main studied effects of sex hormones on the development and progression of GBM, along with principal sex hormone antagonists investigated for their potential antitumor activity. For further details, refer to the text.

Sex Hormone	Estrogens (E2)	Progesterone (P4)	Androgens (Testosterone and Dihydrotestosterone)
Effect on tumorigenesis	- Activation of estrogen receptor α: protumorigenic effects. - Activation of estrogen receptor β: anti-tumorigenic effect [4,89]	Dose-dependent role: pro-tumorigenic effect at lower doses and anti-tumorigenic properties at very high doses [4]	Tumor cell proliferation, migration, and invasion [79]. Induction of changes in the immune microenvironment, promoting GBM immune evasion [80].
Drugs that counteract the effect (potential antitumor agents?)	- 2-methoxyestradiol [97] - Tamoxifen [103]	- RU486 (mifepristone) [112]	- 5α-reductase inhibitors: finasteride and dutasteride [79] - Enzalutamide (androgen receptor inhibitor) ± afatinib [82]

**Table 2:** Sex differences in glioblastoma: hormonal and non-hormonal mechanisms.

Hormonal Mechanisms	Estrogen Receptor α Activation by Estradiol - Promotes tumor cell proliferation, migration, and invasion. Estrogen Receptor β activation by Estradiol: - The protective role of estradiol (E2) in glioma growth and progression [4,89].	Progesterone Receptor Activation: - Stimulates GBM stem cell growth, as well as the infiltration and migration of astrocytes [84,85]. - GBM cells metabolize progesterone (P4) to 5α-dihydroprogesterone (5α-DHP) and further to allopregnanolone (3α-THP), enhancing cell proliferation, migration, cytoprotection, inflammation, survival, invasion, and treatment resistance, potentially bypassing classical P4 receptor pathways [86].	Androgen Receptor Activation by Testosterone and Dihydrotestosterone (DHT): - Promotes tumor cell proliferation, migration, and invasion [79] - Facilitates the infiltration of immunosuppressive regulatory T cells, contributing to immune evasion in GBM [80]. - Enhances EGFR signaling in GBM cells, supporting cell proliferation, survival, and differentiation [75].
Non-Hormonal Mechanisms	Sex Chromosome Contributions: - Two X chromosomes in females allow the expression of genes (e.g., ATRX, DDX3X, and KDM6A) that support the immune response, DNA repair, and cellular growth [116,117]. - A Y chromosome in males plays roles in cell cycle regulation and apoptosis; partial or complete loss is associated with cancer susceptibility, though this is not observed in glioblastoma [118,119].	Immune Response Differences: - Women exhibit stronger immune anti-tumor responses than men, affecting GBM progression [120]. - Sexual dimorphism in microglia and macrophages (GAMs): estrogen induces pro-inflammatory responses in female microglia, while it has anti-inflammatory effects in males [121]. - Sex-specific cytokine production in astrocytes, with males showing higher IL-1β, IL-6, and TNF-α levels, potentially promoting faster GBM growth [122]. Sex-Specific Metabolic Variations: - Differences in glucose utilization for cancer cell growth: men with high glycolytic gene expression have poorer survival, while women with a similar expression show longer survival [124]. - Mitochondrial function varies by sex, with females showing higher enzyme activity (citrate synthase and succinate dehydrogenase) and lower ROS levels, reducing oxidative damage [125].	Molecular and Epigenetic Differences: - Sex-specific molecular variations in GBM: men exhibit the upregulation of oncogenes such as *TP53* and *EGFR* and the downregulation of tumor suppressors like *RB1*, while women display the upregulation of CDK inhibitors and the downregulation of *TP53* [125]. - Sex differences in MGMT promoter methylation: 80% of females vs. 27% of males show hypermethylation, enhancing chemotherapy responses in women [129].

## Data Availability

Not applicable.
